# Expression of iron-related genes in human brain and brain tumors

**DOI:** 10.1186/1471-2202-10-36

**Published:** 2009-04-22

**Authors:** Milla M Hänninen, Joonas Haapasalo, Hannu Haapasalo, Robert E Fleming, Robert S Britton, Bruce R Bacon, Seppo Parkkila

**Affiliations:** 1 Institute of Medical Technology and School of Medicine, University of Tampere, Tampere University Hospital, Tampere, Finland; 2 Department of Pathology, Centre for Laboratory Medicine, Tampere University Hospital, Tampere, Finland; 3 Department of Pediatrics, Saint Louis University Liver Center, Saint Louis University School of Medicine, St. Louis, Missouri, USA; 4 Edward A. Doisy Department of Biochemistry and Molecular Biology, Saint Louis University Liver Center, Saint Louis University School of Medicine, St Louis, Missouri, USA; 5 Division of Gastroenterology and Hepatology, Saint Louis University Liver Center, Saint Louis University School of Medicine, St Louis, Missouri, USA

## Abstract

**Background:**

Defective iron homeostasis may be involved in the development of some diseases within the central nervous system. Although the expression of genes involved in normal iron balance has been intensively studied in other tissues, little is known about their expression in the brain. We investigated the mRNA levels of hepcidin (*HAMP*), HFE, neogenin (*NEO1*), transferrin receptor 1 (*TFRC*), transferrin receptor 2 (*TFR2*), and hemojuvelin (*HFE2*) in normal human brain, brain tumors, and astrocytoma cell lines. The specimens included 5 normal brain tissue samples, 4 meningiomas, one medulloblastoma, 3 oligodendrocytic gliomas, 2 oligoastrocytic gliomas, 8 astrocytic gliomas, and 3 astrocytoma cell lines.

**Results:**

Except for hemojuvelin, all genes studied had detectable levels of mRNA. In most tumor types, the pattern of gene expression was diverse. Notable findings include high expression of transferrin receptor 1 in the hippocampus and medulla oblongata compared to other brain regions, low expression of HFE in normal brain with elevated HFE expression in meningiomas, and absence of hepcidin mRNA in astrocytoma cell lines despite expression in normal brain and tumor specimens.

**Conclusion:**

These results indicate that several iron-related genes are expressed in normal brain, and that their expression may be dysregulated in brain tumors.

## Background

Regulation of iron homeostasis is crucial to maintain normal cell function, and abnormal cellular iron content has been associated with various diseases. Iron is an essential cofactor for many proteins particularly in oxidative reactions and, therefore, neuronal tissues with a high rate of oxidative metabolism have a considerable requirement for iron [1]. The central nervous system (CNS) is not directly in contact with the plasma iron pool, because it resides behind the blood-brain barrier. Like many molecules, the entry of iron is tightly regulated by the blood-brain barrier, and specific transport mechanisms are required to transfer iron into the brain tissue [2]. Several gene products involved in the regulation of iron homeostasis are expressed in the murine CNS, including transferrin receptor 1 (TfR1) [3], iron regulatory protein [4], ferritin [3], neogenin [5], and hepcidin [6]. Even though iron has an essential role for normal physiology, it poses a threat to cells and tissues when present in excess. This feature is based on iron's ability to readily participate in oxidation-reduction reactions and the formation of reactive oxygen intermediates [1]. Iron accumulation has been reported in the nervous tissue of patients suffering from various neurodegenerative disorders, including Alzheimer's disease, Parkinson's disease, amyotrophic lateral sclerosis and age-related macular degeneration [7-10].

Malignant CNS tumors arise worldwide in approximately 189,000 patients per year with an estimated annual mortality of 142,000 [11]. Malignant gliomas, including the most common subtype, glioblastoma, are among the most devastating neoplasms and still pose a great challenge for both diagnosis and treatment. Malignant gliomas derive from glial cells and exhibit aggressive tumor characteristics such as high proliferation rate, diminished apoptosis, and escape from external growth control. A distinctive feature of gliomas is that they very rarely metastasize beyond the CNS, despite their highly invasive and angiogenic capabilities [12].

In neoplastic tissues, the oxygen requirement is high, and thus angiogenesis is often crucial for cancer survival. For malignant CNS tumors, especially gliomas, neovascularization is directly correlated with their biological aggressiveness, degree of malignancy and clinical recurrence, and is inversely correlated with post-operative survival time [13]. Neovascularization is driven by a complex regulatory pathway involving von Hippel-Lindau protein (pVHL) and hypoxia-inducible factor (HIF). In normoxia, the transcription factor HIF is targeted for proteosomal degradation, whereas it is stabilized in hypoxic conditions. HIF accumulation induces the expression of several proangiogenic proteins, including transferrin and TfR1, that are linked to the regulation of cellular iron homeostasis [13].

Hepcidin is a key regulator of iron homeostasis that is abundantly expressed in the liver [14,15]. Iron overload leads to increased hepcidin synthesis, while anaemia, hypoxia, and inflammation downregulate its expression [16]. A recent study showed that hypoxia downregulates hepcidin expression via the VHL/HIF pathway [17]. Hepcidin acts by modulating the activity of ferroportin, the only iron export protein known to exist in mammalian cells. By binding to ferroportin, hepcidin causes its internalization and degradation and, thereby, decreases cellular iron export [18]. Hemojuvelin and its putative co-regulator, neogenin, are other factors that may be involved in the regulation of hepcidin expression [19-21].

Because malignant cells have a high metabolic rate and require significant quantities of iron for various cellular processes, our hypothesis was that iron related genes are differentially expressed in brain tumor cells versus normal brain tissue. The goal of this study was to investigate the expression levels of genes related to iron homeostasis in both brain tumors and normal brain regions. The mRNA expression levels of hepcidin (*HAMP*), HFE, neogenin (*NEO1*), transferrin receptor 1 (*TFRC*), transferrin receptor 2 (*TFR2*), and hemojuvelin (*HFE2*) were measured. We found that all the studied genes, except for hemojuvelin, were expressed in normal brain, and altered expression patterns were found in brain tumors and astrocytoma cell lines. The results suggest that these genes might play a role in the maintenance of iron homeostasis in the brain, and their differential expression in brain tumors may contribute to tumor pathogenesis.

## Methods

### Study materials for RNA isolation

The study material consisted of: 1) 18 brain tumors obtained as histopathology samples from surgical patients in Tampere University Hospital, Tampere, Finland between 1983 and 2001 (Table 1); 2) 5 samples of normal brain tissue, and 3) 3 astrocytoma cell lines. The experiments were approved by the Ethical Committee of the Tampere University Hospital and conducted according to the guidelines of the Helsinki Declaration. The brain tumors comprised 4 meningiomas (2 grade II; 2 grade III), 3 oligodendrocytic gliomas (2 grade II; 1 grade III), 2 oligoastrocytic gliomas (1 grade II; 1 grade III), one medulloblastoma (grade IV), one pilocytic astrocytoma (grade I), and 7 diffusively infiltrating astrocytic gliomas (2 grade II; 4 grade III; 1 grade IV). The age of the patients ranged from 19 to 86 years (48 ± 18, mean ± SD). Normal brain tissue was obtained post-mortem upon autopsy, and included samples of cortex, cerebellum, thalamus, medulla oblongata, and hippocampus. The cell lines, CCF-STTG1, U373 MG, and U-87 MG, were originally derived from grade IV astrocytomas (glioblastomas). These cell lines were obtained from the European Collection of Cell Cultures (Salisbury, UK) and were cultured according to the provider's instructions.

**Table 1:** Patient information for brain tumor samples

**Patient**	**Type of tumor**	**Stage**	**Age at diagnosis**	**Sex**
1	Meningioma	I	72	F
2	Meningioma	I	62	F
3	Meningioma	II	86	F
4	Meningioma	II	27	M
				
5	Oligodendroglioma	II	54	F
6	Oligodendroglioma	II	47	M
7	Oligodendroglioma	III	59	M
				
8	Oligoastrocytoma	II	44	F
9	Oligoastrocytoma	III	40	M
				
10	Medulloblastoma	IV	19	F
				
11	Astrocytoma	I	21	M
12	Astrocytoma	II	26	M
13	Astrocytoma	II	44	F
14	Astrocytoma	III	44	M
15	Astrocytoma	III	51	F
16	Astrocytoma	III	61	F
17	Astrocytoma	III	44	M
18	Astrocytoma	IV	62	M

### Statistical analysis

Spearman's rank correlation coefficient was used to determine the influence of age on expression levels. The results were considered statistically significant when the correlation coefficient was < -0.300 or > 0.300, with p-value < 0.05. Mann-Whitney U test was used to evaluate the effect of sex on the expression levels. The results were considered statistically significant when p-value was < 0.05.

### RNA isolation and first-strand cDNA synthesis

All tissue specimens to be used for RNA isolation were placed in RNAlater (Ambion, Austin, TX) and stored at -80°C until use. Total RNA was isolated from tissue samples using QIAzol lysis reagent and RNeasy Lipid Tissue Mini Kit (Qiagen, Germantown, MD) and from cell cultures using RNeasy Mini Kit (Qiagen) following the manufacturer's instructions. Both protocols included treatment with RNase-free DNase I (Novagen, Madison, WI). RNA concentration and purity were determined by optical density measurement at 260 and 280 nm. All the samples had an OD260/OD280 ratio of 2.0 or higher. For each sample, 0.6 μg of total RNA were converted into first-strand cDNA using the First-Strand cDNA Synthesis Kit (Fermentas, Burlington, Canada) and oligo d(T) primers according to the protocol recommended by the manufacturer.

### Reverse transcription PCR and quantitative real-time PCR

The relative amounts of human *HAMP*, *HFE*, *NEO1*, *TFRC*, and *TFR2 *transcripts in different tissues were first assessed by reverse transcription (RT) PCR and then with quantitative real-time PCR using the LightCycler detection system (Roche, Rotkreuz, Switzerland). *HFE2 *expression was studied only by RT-PCR which remained completely negative. The RT-PCR and the real-time PCR primers for the target transcripts were designed using the complete cDNA sequences deposited in GenBank (accession numbers: NM_000410 for *HFE*, NM_021175 for *HAMP*, NM_003234 for *TFRC*, NM_003227 for *TFR2*, NM_002499 for *NEO1 *and NM_213653 for *HFE2*). To avoid amplification of contaminating genomic DNA, the two primers from each primer set were placed in different exons. *HPRT1 *(hypoxanthine phosphoribosyl-transferase I), *GAPDH *(glyceraldehyde-3-phosphate dehydrogenase), *TBP *(TATA box binding protein), and *UBC *(ubiquitin C) were used as internal controls to normalize for potential quality and quantity differences between samples. According to study of Vandesompele et al. four internal control genes are required for accurate normalization of the samples [22]. The internal control transcripts and primer sequences for HPRT1, GAPDH, UBC were chosen based on the results of Vandesompele et al. [22]. Table 2 shows the primer sequences used to measure the internal control transcripts and the target transcripts.

**Table 2:** Primer sequences for quantitative real-time PCR

**Gene**	**Forward primer (5'-3')**	**Reverse promer (5'-3')**
HPRT1*	TGACACTGGCAAAACAATGCA	GGTCCTTTCACCACAAGCT
GAPDH*	TGCACCACCAACTGCTTAGC	GGCATGGACTGTGGTCATGAG
UBC*	ATTTGGGTCGCGGTTCTTG	TGCCTTGACATTCTCGATGGT
TBP	TGCACAGGAGCCAAGAGTGAA	CACATCACAGCTCCCCACCA
HFE	CAACAAGTGCCTCCTTTGGT	GGGGGTACAGCCAAGGTTAT
HAMP	CCACAACAGACGGGACAAC	CTTGCAGCACATCCCACAC
NEO1	GCTGGAAGGCGAGGAATGA	CGGGTGGTAGAGATGACTG
TFRC	CTTTGGACATGCTCATCTGG	TGTTTTCCAGTCAGAGGGACA
TFR2	TGGAGGACACCATCAGGCAA	AGCTGCTCTCCGACCTTCC
HFE2**	GGAGAATTGGATAGCAGAG	AGCAGCAGGAGAGTGAGA
B2M**	TCCAGCGTACTCCAAAGATTCAGG	ATGCGGCATCTTCAAACCTCC

* Primer sequences first described in [22]

** Primers used for RT-PCR only

PCR reactions were performed using ReddyMix PCR Master Mix (ABgene, UK) according to manufacturer's instructions. *B2M *(beta-2-microglobulin) was used as positive control for the RT-PCR reactions. The PCR products were visualized by gel electrophoresis in 1.5% agarose gel with 0.1 μL/mL ethidium bromide. Quantitative real-time PCR reactions were carried out in a 20 μL volume containing 0.5 μL of first strand cDNA, 1× of QuantiTect SYBR Green PCR Master Mix (Qiagen, Hilden, Germany), and 0.5 μM of forward and reverse primers. Amplification and detection were carried out as follows. After an initial 15-min activation step at 95°C, 45 cycles consisting of denaturation at 94°C, 15 s; annealing at Tm, 20 s; elongation at 72°C, 20 s, and final cooling step. Melting curve analysis was always performed after the amplification to check PCR specificity. To quantify the concentration of both the internal control transcripts and the transcripts for *HAMP*, *HFE*, *NEO1*, *TFRC*, and *TFR2 *in the samples, a standard curve for each transcript was established using 5-fold serial dilutions of known concentrations of purified PCR products generated from the same primer sets. Each cDNA sample was tested in triplicate. Replicate analyses were automatically handled by the software of the real-time PCR machine. The crossing point value obtained was utilized to determine the amount of original transcript using the specific standard curve. The geometric mean of the 4 internal control transcripts was used as a normalization factor for gene expression levels [22]. The final relative mRNA expression was designated as the copy number of a target transcript in each tissue divided by the corresponding normalization factor and subsequently multiplied by 10^2 ^or 10^3^.

## Results and discussion

In this study, the mRNA expression levels of hepcidin (*HAMP*), HFE, neogenin (*NEO1*), TfR1 (*TFRC*), transferrin receptor 2 (*TFR2*), and hemojuvelin (*HFE2*) were examined in normal human brain tissue, brain tumors, and astrocytoma cell lines. Hemojuvelin showed no expression in RT-PCR experiments and, therefore, it was not analyzed further by quantitative real-time PCR.

The statistical analysis revealed no significant influence of either age or sex on the expression levels of genes studied.

**Figure 1:**
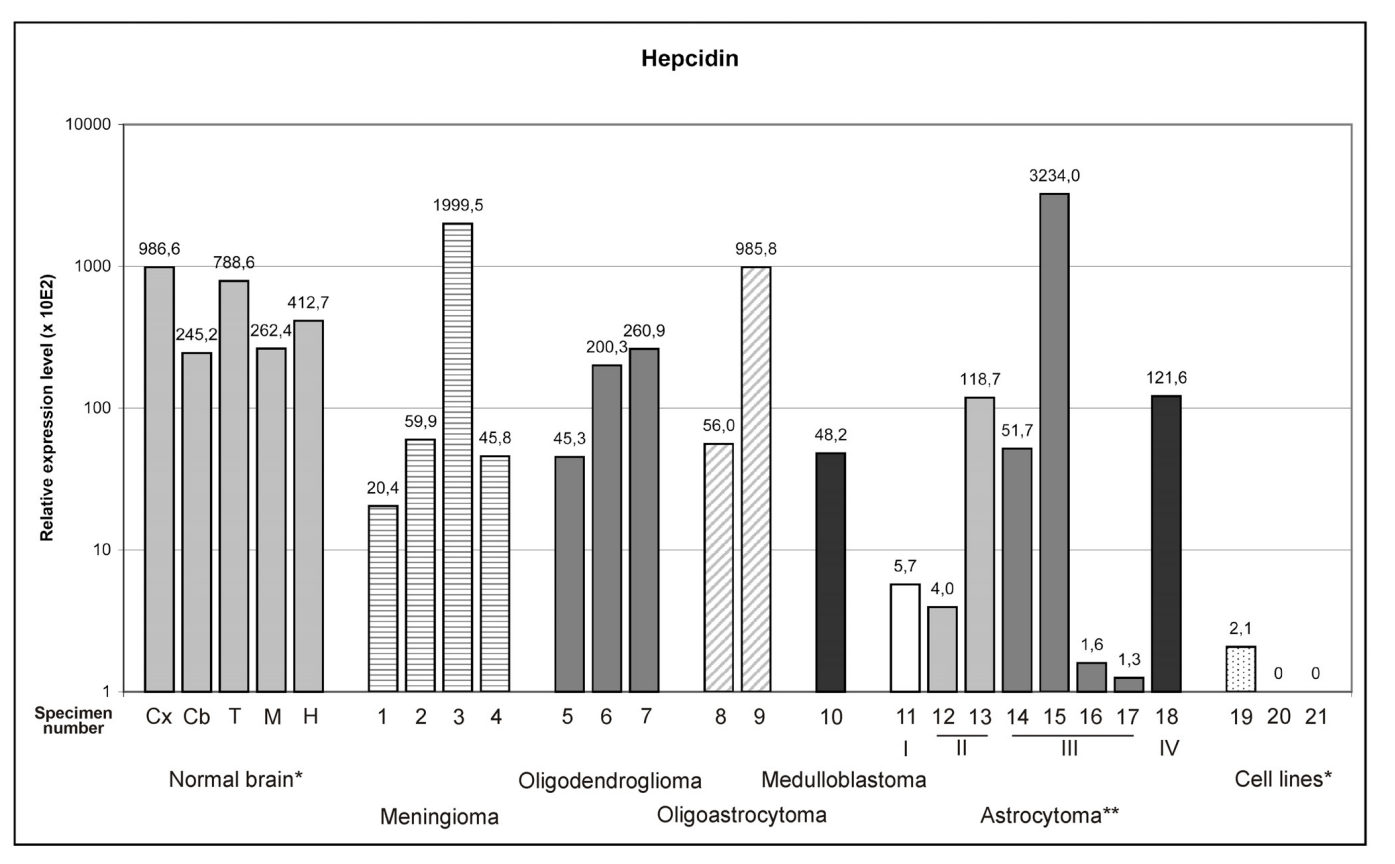
**Hepcidin mRNA expression in normal brain and brain tumors detected by real-time PCR**. The expression is normalized using internal control genes and relative expression levels are shown. *Abbreviations used for normal brain: Cx = cortex, Cb = cerebellum, T = thalamus, M = medulla oblongata, H = hippocampus. Tumor samples are numbered according to Table 1. Numbers 19 to 21 are used to indicate cell lines: 19 = CCF-STTG1, 20 = U373MG, 21 = U-87 MG. **Roman numbers in astrocytomas indicate tumor grade.

Hepcidin (*HAMP*) showed relatively high expression levels in all brain regions examined (Fig. 1); the highest expression was found in the cortex and thalamus. Previously, hepcidin expression has been demonstrated in murine brain [6], but it appears to be absent in the catfish brain [23]. Taken together, these results indicate that hepcidin mRNA is produced in mammalian brain tissue where the encoded hepcidin peptide may contribute to iron homeostasis as well as to mechanisms protecting the CNS from microbial infections. Most tumor samples had lower expression of hepcidin compared to normal brain cortex, and the levels were negligible in the astrocytoma cell lines (Fig. 1). The absence of hepcidin mRNA in the astrocytoma cell lines was surprising because it was expressed in most tissue specimens. One explanation could be that some non-neuronal/non-glial cells such as endothelial cells might be the predominant site of hepcidin expression in the brain. However, this is unlikely because Zechel et al. [6] have reported hepcidin expression in both neurons and glia cells. It is important to realize that the cell lines were cultured in optimized and normoxic conditions, while the hypoxia is the key regulator of variable genes in tumors growing in the hostile environment [13,24]. Although the exact molecular mechanisms remain unclear, intratumoral factors including paracrine regulation might contribute to the unexpected expression pattern of hepcidin. Cultured cells may be lacking some key factors which induce intratumoral hepcidin expression. Previous studies have identified several factors that regulate hepcidin mRNA expression in the liver. For example, iron overload increases hepcidin expression, whereas anemia and hypoxia lead to downregulation [25]. Hypoxia acting through a VHL/HIF-induced pathway induces the expression of several genes in tumors which, in turn, may increase the resistance of tumor cells to their hostile microenvironment [26,27]. Peyssonnaux et al. [17] recently showed that a VHL/HIF pathway regulates hepatic hepcidin expression *in vivo*, but the potential role of this pathway in modulating brain hepcidin is unknown.

Hemojuvelin also plays an important role in hepcidin regulation in the liver, acting as a co-receptor for bone morphogenetic protein [20]. Hemojuvelin exists in both membrane-bound and soluble forms, and Silvestri et al. [28] have demonstrated regulation of soluble hemojuvelin via HIF-1α and furin. However, our results indicate that hemojuvelin mRNA is not expressed in normal brain tissue or in brain tumors. A previous study demonstrated that murine brain also does not express hemojuvelin [5]. Taken together, the available data suggest that hemojuvelin may not regulate hepcidin expression in the brain, or soluble hemojuvelin produced elsewhere is transferred across the blood-brain barrier to regulate hepcidin expression in the brain.

**Figure 2:**
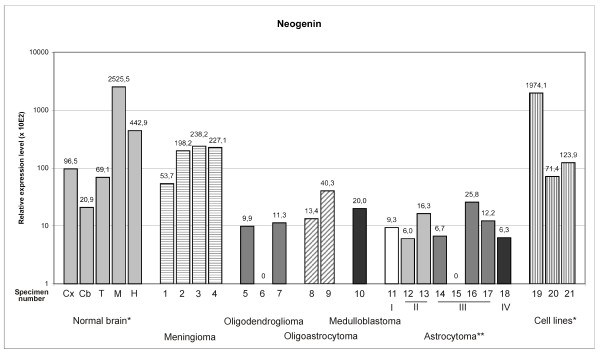
**Neogenin mRNA expression in normal brain and brain tumors detected by real-time PCR**. The expression is normalized using internal control genes and relative expression levels are shown. *Abbreviations used for normal brain: Cx = cortex, Cb = cerebellum, T = thalamus, M = medulla oblongata, H = hippocampus. Tumor samples are numbered according to Table 1. Numbers 19 to 21 are used to indicate cell lines: 19 = CCF-STTG1, 20 = U373MG, 21 = U-87 MG. **Roman numbers in astrocytomas indicate tumor grade.

Neogenin has been demonstrated to interact with hemojuvelin, and it has been hypothesized that, through this interaction, neogenin may influence hepcidin expression [19,21,29]. A previous report has shown that neogenin is expressed widely in murine tissues, including the brain [5]. We observed that neogenin mRNA is abundant in normal human brain tissue, and the highest expression levels were found in the medulla oblongata and hippocampus (Fig. 2). In contrast, neogenin mRNA levels were low in oligodendrogliomas, oligoastrocytomas, medulloblastomas, and astrocytomas, while in meningiomas and astrocytoma cell lines, neogenin mRNA expression is within the same range as seen in normal brain regions. This diverse expression pattern in brain tumors is not unexpected because both overexpression and repression of neogenin have been previously reported in neoplastic tissues [30,31]. The function of neogenin in the adult brain tissue remains to be determined.

**Figure 3:**
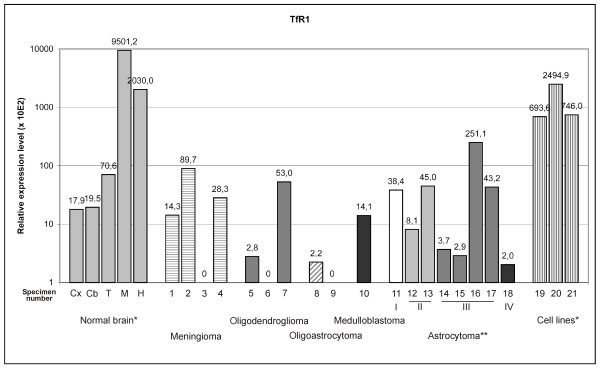
**Transferrin receptor 1 mRNA expression in normal brain and brain tumors detected by real-time PCR**. The expression is normalized using internal control genes and relative expression levels are shown. *Abbreviations used for normal brain: Cx = cortex, Cb = cerebellum, T = thalamus, M = medulla oblongata, H = hippocampus. Tumor samples are numbered according to Table 1. Numbers 19 to 21 are used to indicate cell lines: 19 = CCF-STTG1, 20 = U373MG, 21 = U-87 MG. **Roman numbers in astrocytomas indicate tumor grade.

**Figure 4:**
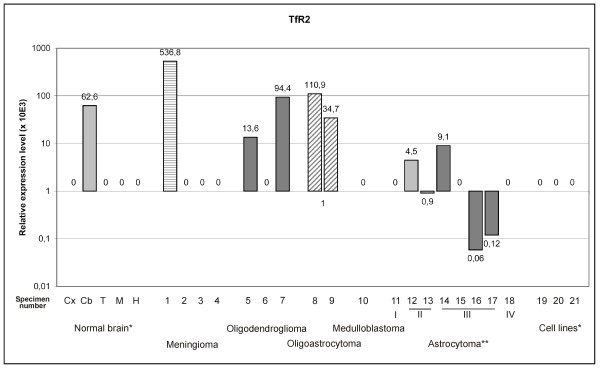
**Transferrin receptor 2 mRNA expression in normal brain and brain tumors detected by real-time PCR**. The expression is normalized using internal control genes and relative expression levels are shown. *Abbreviations used for normal brain: Cx = cortex, Cb = cerebellum, T = thalamus, M = medulla oblongata, H = hippocampus. Tumor samples are numbered according to Table 1. Numbers 19 to 21 are used to indicate cell lines: 19 = CCF-STTG1, 20 = U373MG, 21 = U-87 MG. **Roman numbers in astrocytomas indicate tumor grade.

In the brain, the highest expression levels for TfR1 mRNA were in the medulla oblongata and the hippocampus, and expression was distinctly lower in the cortex, thalamus, and cerebellum (Fig. 3). The brain tumors had no differential expression of TfR1 mRNA compared to normal human cortex. However, the astrocytoma cell lines had substantially higher TfR1 expression than the brain tumors. These differences in TfR1 expression levels agree with the expression intensities and patterns observed in a previous study [32]. The cerebellum was the only part of the normal brain expressing TfR2 mRNA (Fig. 4). Some TfR2 mRNA expression was present in a few brain tumor specimens, with possible differential expression in oligoastrocytomas. TfR2 mRNA was not expressed in astrocytoma cell lines.

**Figure 5:**
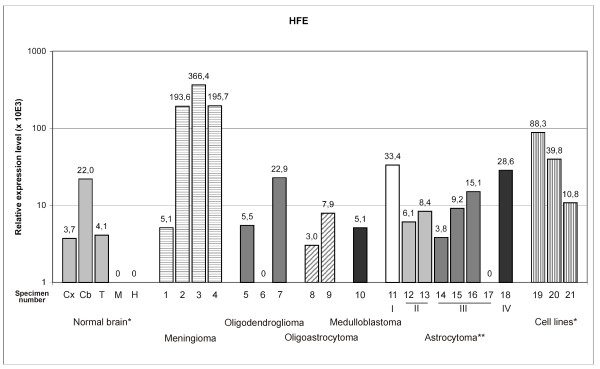
**HFE mRNA expression in normal brain and brain tumors detected by real-time PCR**. The expression is normalized using internal control genes and relative expression levels are shown. *Abbreviations used for normal brain: Cx = cortex, Cb = cerebellum, T = thalamus, M = medulla oblongata, H = hippocampus. Tumor samples are numbered according to Table 1. Numbers 19 to 21 are used to indicate cell lines: 19 = CCF-STTG1, 20 = U373MG, 21 = U-87 MG. **Roman numbers in astrocytomas indicate tumor grade.

The *HFE *gene encodes a MHC type 1-like protein that is involved in modulating iron homeostasis [33,34]. We observed that HFE mRNA was expressed at low levels in human cortex, cerebellum, and thalamus, but not in medulla oblongata or hippocampus (Fig. 5). Brain tumors and astrocytoma cell lines showed somewhat higher expression of HFE mRNA than normal cortex. The highest mRNA levels were present in 3 out of 4 meningioma specimens. Mutations in the *HFE *gene result in the most common form of hereditary hemochromatosis. Interestingly, some studies suggest that one of the most common *HFE *mutations, H63D, may be a genetic modifier for increased risk of Alzheimer's disease and amyotrophic lateral sclerosis [35-37].

Although investigation of the molecular basis of iron homeostasis has been intensive after the discovery of the *HFE *gene in 1996 [34], little is still known about the role of iron and iron-related genes in the brain. In this study, we show that many of the genes considered essential for the regulation of iron homeostasis outside the CNS are also expressed in the brain. Iron regulation in the brain is complex, and in future studies it will be of great interest to investigate the protein expression of these molecules in different brain regions along with assessment of regional iron concentrations. Due to the presence of the blood-brain barrier, the CNS can adjust its iron status somewhat independently of other organs. Our results also indicate that the expression pattern of iron-related genes can vary markedly in tumors arising within the brain. Further investigation will be required to evaluate whether the dysregulated expression of iron-related genes in brain tumors may contribute to tumor pathogenesis.

## Conclusion

The present study describes the expression of several iron-related genes including hepcidin (*HAMP*), HFE, neogenin(*NEO1*), transferrin receptor 1 (*TFRC*), transferrin receptor 2 (*TFR2*), and hemojuvelin(*HFE2*) in normal human brain, brain tumors, and astrocytoma cell lines. Our results suggested that all these genes except for *HFE2 *are expressed in the normal brain, and that their expression may be dysregulated in certain brain tumors.

## Authors' contributions

SP, RSB, REF, and BRB designed the study. JH and HH collected the tissue specimens. MMH cultured the cells and performed the real-time PCR. MMH drafted the manuscript. All authors revised and approved the final manuscript.
